# Screening participants with inflammatory bowel disease or high colorectal cancer risk in Denmark: a cohort study

**DOI:** 10.1057/s41271-024-00523-z

**Published:** 2024-10-16

**Authors:** Signe Bülow Therkildsen, Pernille Thordal Larsen, Sisse Helle Njor

**Affiliations:** 1 Department of Public Health Programmes, University Research Clinic for Cancer Screening, Randers Regional Hospital, Skovlyvej 15, 8930 Randers NØ, Denmark; 2 Department of Clinical Medicine, Aarhus University, Palle-Juul-Jensen Boulevard 82, 8200 Aarhus N, Denmark; 3 Research Unit for Screening and Epidemiology, Department for Biochemistry and Immunology, University Hospital of Southern Denmark, Vejle, Beriderbakken 4, 7100 Vejle, Denmark

**Keywords:** FIT screening, Colorectal cancer screening, Inflammatory bowel disease, False-positive, Recommendations, High-risk individuals

## Abstract

Individuals with inflammatory bowel disease (IBC) and high-risk individuals are advised to discuss participation with their doctor and not to participate in colorectal cancer (CRC) screening. Yet a substantial proportion still participate in the Danish faecal immunochemical test (FIT) screening and have a higher positive FIT rate than the average-risk population. We estimated the risk of false-positive screening among individuals with inflammatory bowel disease and high-risk individuals to improve recommendations regarding screening participation. We included 71,871 FIT-positive participants (2014–2017) who had a subsequent colonoscopy within 3 months. Screening outcome within 180 days was established by using registers. We determined that 26,591 of the included participants had a false-positive screening. Participants with IBC or high CRC risk had a significantly higher risk of getting a false-positive screening than the average risk population, resulting in too many screening-related colonoscopies being performed among these individuals, indicating a need to update the screening protocols.

**Supplementary Information:**

The online version contains supplementary material available at 10.1057/s41271-024-00523-z.

## Key messages

- Individuals not recommended colorectal cancer screening have a higher false-positive risk.
- Thereby too many screening-advised colonoscopies are performed in the Danish colorectal cancer screening programme.
- Updating the information in the invitation letter might avoid redundant procedures.

## Introduction

In 2003 the European Council recommended implementation of colorectal cancer (CRC) screening [1]. Since then, CRC screening has been part of the public health policy widely in the European Union [2]. The faecal immunochemical test (FIT) based CRC screening program is developed for screening of men and women aged 50–74 years who have an average CRC risk and no previous CRC diagnosis [3, 4]. High-risk individuals with a previous CRC diagnosis or polyps associated with hereditary CRC syndromes are not recommended to participate in FIT screening, as they should instead be enrolled in a surveillance program [5, 6]. Several studies have investigated whether individuals with inflammatory bowel diseases (IBD), such as Crohn's disease (CD) and ulcerative colitis (UC), have a higher risk of developing CRC compared to individuals without these diseases [7–9]. It has been shown that subgroups of individuals with IBD are at higher risk of developing CRC, mainly individuals with UC [10]. Furthermore, FIT has been shown to be an effective biomarker for disease activity for individuals with IBD [11–13]. Therefore, the invitation letter to FIT screening in Denmark, recommend individuals with IBD to discuss participation with their doctor [4, 14]. Individuals with long-time IBD, post-inflammatory polyps, colon or rectal dysplasia or primary sclerosing cholangitis (PSC) undergo continuous colonoscopy monitoring, with frequency ranging from every three months to every five years based on risk factors[15], wherefore these individuals should not be recommended to participate in FIT screening.

We have shown that despite this, 29% of the individuals with a previous CRC diagnosis, 48% of the individuals with multiple/hereditary polyp syndromes, and 55% of the individuals with IBD chose to participate in the Danish FIT screening program when invited [4]. In addition, participants with multiple and hereditary polyp syndromes and participants with IBD had a significantly higher positive FIT rate than the average-risk population [4]. However, to our knowledge, it is unknown whether the higher FIT-positive rates among these groups are due to a higher risk of detecting CRC or precursors to CRC in the following colonoscopies or whether it is a result of a higher number of false-positive cases. It has recently been shown that IBD is an independent risk factor for false-positive results in the FIT-based CRC screening in Germany [16].

This study aims to investigate the outcome of the screen-derived colonoscopies and the risk of false-positive screening among participants who are not in the target population for the Danish National CRC screening program. This information might help to improve recommendations regarding FIT-based CRC screening participation of high-risk individuals and individuals with IBD.

## Data and methods

### Study setting

The Danish FIT-based CRC screening program was implemented in 2014. All residents aged 50–74 years on 1 January 2014 were invited to participate during 2014–2017. Since 2018, FIT screening has been offered every two years to men and women aged 50–74 years [14]. The Danish CRC screening program, as well as subsequent procedures and treatments is free of charge and a part of the tax-funded Danish health care system [17]. Invitations are sent by mail and include an information letter, the FIT test kit: a sample bottle, faeces collection sheet, and the OC Sensor FIT [14]. The returned FIT sample is considered positive if it contains ≥ 20 µg haemoglobin/g faeces. Participants with a positive FIT test are by law invited to a colonoscopy within two weeks. Based on the colonoscopy, FIT-positive residents are categorized with either: clean colon, low-risk adenomas, intermediate-risk adenomas, high-risk adenomas, or CRC [14, 18, 19]**.**

### Study design and population

This study is a nationwide register-based cohort study. We included all participants aged 50–74 years who had a positive index FIT, within six months after receiving an invitation to the Danish national CRC screening program, between 1 January 2014 and 31 December 2017. The included participants were grouped into the following categories: (1) average-risk participants, (2) participants with previous CRC, (3) participants with multiple/hereditary polyp syndromes, (4) participants with UC, (5) participants with CD and (6) participants with UC and CD.

Participants not belonging to groups 2–6, who in the 10 years preceding the screening invitation had colorectal polyps were excluded from the average-risk group, to ensure that the group did not include participants under surveillance. Participants with PSC and either UC or CD were included in the respective group. Participants who did not participate in the subsequent colonoscopy within three months after their positive FIT were excluded from the main analysis. Participants were followed for 180 days after the colonoscopy, to find the outcome of the subsequent colonoscopy.

The outcome of the screen-derived colonoscopy after a positive FIT was either clean colon, low-risk adenomas, intermediate-risk adenomas, high-risk adenomas or CRC [14, 19]. We dichotomized the outcome of the colonoscopy to categorize the screening outcome, so participants with clean colon were coded as having a false-positive screening, whereas participants with low-risk, intermediate-risk, high-risk, or CRC were coded as having a true-positive screening.

All Danish residents have a unique civil registration number (CRN) in the Danish civil registration system. Information on sex and date of birth was obtained from the Danish civil registration system [20]. The CRN was merged with several Danish registers: The Danish Colorectal Cancer Screening Database (DCCSD) [21], The Danish Cancer Registry (DCR) [22], the Danish Colorectal Cancer Group (DCCG) Database [23], the Danish National Patient Register (DNPR) [24], and the Danish Pathology Registry (DPR) [25].

The invitation date and the date of the positive FIT were obtained from DCCSD. The purpose of the database is to monitor the quality of the screening program and DCCSD gets information from the administrative database IAM [21]. Information on CRC diagnosis, based on the Danish version of the International Classification of Diseases (ICD), was obtained from DCR and DCCG [22, 23]. Information on IBD and multiple/hereditary polyp syndromes, based on ICD, were obtained from DNPR, together with dates of performed colonoscopies based on procedure codes (KUJF32, KUJF35) [24]. Detailed codes used to define the exposure groups are presented in Larsen et al. [4].

The outcome of the colonoscopy examination was based on obtained information on CRC from DCR and DCCG [22, 23] and polyps from DNPR and DPR (DK-snomed: M8213F, M82110, M82130, M8213M, M82630, M82611, M8213s, and M72040) including information on numbers, size, and grade of neoplasia [24, 25]. We used the following procedure codes from DNPR: clean colon (AFX02C or AFX02D), low-risk adenomas (ZPY1E03), intermediate-risk adenomas (ZPY1E02) and high-risk adenomas (ZPY1E01)[24]. The risk codes and information about cancer were cross-checked with data from DPR [25], to ensure the most accurate recording of the colonoscopy outcome. The categorization was based on the highest possible risk group, where pathology data were given the highest weight. All malignant findings from the colon and rectum were included, also malignant findings in the colon or rectum arising from cancer elsewhere. Detailed definition of risk groups based on colonoscopy outcome is described in Table 1.

**Table 1:** Definition of risk groups

Colonoscopy outcome	Definition	Codes
Clean colon	No finding of sessile polyps, adenomas or tumour	AFX02C or AFX02D from DNPR
Low-risk	1–2 adenomas < 10 mm	ZPY1E01from DNPR or DK-snomed codes in DPR specifying that it is low-risk adenomas
	Sessile serrated lesions < 10 mm	
	Low grade neoplasia	
Intermediate-risk	3–4 adenomas or 1 adenoma/sessile serrated lesions ≥ 10 mm. and < 20 mm	ZPY1E02 from DNPR DK-snomed codes in DPR specifying that it is medium-risk adenomas
	Tubulo-villous or villous adenoma	
	High grade neoplasia	
High-risk	≥ 5 adenomas or 1 adenoma ≥ 20 mm	ZPY1E03 from DNPR or DK-snomed codes in DPR specifying that it is high-risk adenomas
	Removal of adenomas with piecemeal technique	
Colorectal cancer	Malignant findings in the colorectum	DC18, DC19 or DC20 from DCR and/or DCCG

### Statistical analysis

We calculated the proportion of FIT-positive participants who attended the colonoscopy within three months among the six groups (average-risk, previous CRC diagnosis, multiple and hereditary polyp syndromes, UC, CD, and UC and CD). The median age and the proportion of men are also presented for each of the six groups. We estimated the odds of having a false-positive screening test for the various groups relative to average-risk participants. We used multiple logistic regression to adjust for sex (male or female) and five year-age groups (50–54 years, 55–59 years, 60–64 years, 65–69 years, and 70–74 years). Participants with unknown registration of the colonoscopy outcomes were excluded from this analysis. The proportions of colonoscopy outcomes (clean colon, low-risk, intermediate-risk, high-risk, CRC, and unknown registration) are presented for each group, respectively. Due to a small number of participants with multiple and hereditary polyp syndromes as well as both UC and CD, these proportions are only presented for average-risk participants and participants with a previous CRC diagnosis, UC, or CD.

All analyses were performed in STATA statistical software version 17 (Stata Corp, College Station, TX). Estimates are presented with 95% confidence intervals (CI) and a statistically significant result was defined as a *p*-value < 0.05.

### Ethical considerations

According to the Danish Legislation and the Central Denmark Region Committees on Biomedical Research ethics, this study did not require ethical approval, as the entire study was only based on registry data. The project was listed in the record of processing activities for research projects in the Central Denmark region (J. No-:1-16-02-429-19) according to the EU's General Data Protection Regulation.

## Results

In total 78,575 participants were registered with a positive index FIT in the Danish CRC screening program between 1 January 2014 and 31 December 2017. We excluded 6704 (8.5%) participants who did not attend the subsequent colonoscopy within three months. A higher proportion of the average-risk group attended the subsequent colonoscopy compared to participants not in the target population for FIT-based CRC screening. The proportions of participants within the six groups are shown in Table 2.

**Table 2:** Proportion of FIT positive who attended colonoscopy within three months

Group	Positive index FIT N	Attended colonoscopy n (%)
	*N* = 78,575	N = 71,871 (91.5%)
Average risk	75,978	69,892 (92.0)
Prior colorectal cancer	444	361 (81.3)
Multiple/hereditary polyp syndromes	70	51 (72.9)
Ulcerative colitis	1,466	1,100 (75.0)
Crohn's disease	403	317 (78.7)
Ulcerative colitis and Crohn's disease	214	150 (70.1)

In total 71,871 attended the following colonoscopy and were eligible for further analysis. The sex and age composition within the groups are shown in Table 3. The median age was highest among participants with previous CRC and lowest among participants with IBD. Overall, the proportion of men was higher than women, except for participants with CD.

**Table 3:** Mean age and proportion of men within the five groups

Group	Attended colonoscopy	Age median (IQR)	Men *n* (%)
Average risk	69,892	65.2 (12.6)	38,969 (55.8)
Prior colorectal cancer	361	70.3 (7.6)	210 (58.2)
Multiple/hereditary polyp syndromes	51	69.1 (7.1)	35 (68.6)
Ulcerative colitis	1,100	62.5 (13.3)	586 (53.3)
Crohn's disease	317	62.4 (14.0)	141 (44.5)
Ulcerative colitis and Crohn's disease	150	61.3 (14.1)	77 (51.3)

Due to unknown registration of the colonoscopy outcome, 1898 participants could not be categorized as having either a true or a false-positive screening. Among the remaining 69,973 participants, 38% were registered with a false-positive screening. The median age of the participants with a false-positive screening was 63.4 years (IQR:14.5) and the proportion of men was 47.3%. Among the participants with a true-positive screening, the median age was 66.2 years (IQR:11.6) and the proportion of men was 61.3%. Participants with CRC, UC, CD, and both UC and CD had a statistically significantly higher proportion of false-positive screening compared to the average-risk group, even after adjusting for age and sex. Participants with both UC and CD had the highest odds of a false-positive screening compared to the average-risk group (OR adjusted: 3.83 (95%CI: 2.59;5.56)) while participants with multiple/hereditary polyp syndromes had the lowest odds of false-positive screening compared to the average-risk group [OR adjusted: 0.63 (95%CI: 0.33;1.23)] (Table 4).

**Table 4:** Proportion and odds of false-positive relative to average-risk participants

Group	Attended colonoscopy N**	False positive *n* (%)	OR (95%CI)	Adjusted* OR (95%CI)
Average risk	68,141	25,435 (37.3)	1	1
Prior colorectal cancer	338	155 (45.9)	1.42 (1.15; 1.76)	1.68 (1.35;2.09)
Multiple/hereditary polyp	> 40	12 (NA)	0.54 (0.28;1.04)	0.63 (0.33;1.23)
Ulcerative colitis	1026	712 (69.4)	3.81 (3.33;4.35)	3.75 (3.27;4.30)
Crohn's disease	287	184 (64.1)	3.00 (2.36;3.82)	2.72 (2.13;3.49)
Ulcerative colitis and Crohn's disease	133	93 (70.5)	4.00 (2.75;5.82)	3.80 (2.59;5.56)

*Adjusted for sex and five-year age groups

**Participants with unknown registration of colonoscopy outcome are not included in this analysis

The outcome of the colonoscopy among FIT-positive participants (average-risk, CRC, UC, or CD) can be seen in Table 5. The proportion of clean colon varied from 36.4 to 64.7% among the groups and was highest among participants with UC and lowest among average-risk participants. The proportion who had CRC detected at the colonoscopy varied from 1.4 to 6.1% and was highest among the average-risk participants and lowest among participants with UC. There was a higher number of participants with CD (9.5%), UC (6.7%), and CRC (6.4%) that had unknown registration, compared to the average-risk participants (2.5%).

**Table 5:** Colonoscopy outcome among participants within the groups; average-risk, prior cancer, Ulcerative colitis and Crohn's disease

Group	Outcome of colonoscopy examination n (%)					
	Clean colon *N* = 26.486	Low-risk *N* = 14.450	Intermediate-risk *N* = 11.780	High-risk *N* = 12.777	Cancer *N* = 4.299	Unknown *N* = 1.878
Average	25.435 (36.4)	14.161 (20.3)	11.634 (16.7)	12.657 (18.1)	4.254 (6.1)	1.751 (2.5)
Prior cancer	155 (42.9)	76 (21.1)	51 (14.1)	38 (10.5)	18 (5.0)	23 (6.4)
Ulcerative colitis	712 (64.7)	169 (15.4)	66 (6.0)	64 (5.8)	15 (1.4)	74 (6.7)
Crohn's disease	184 (58.0)	44 (13.9)	29 (9.2)	18 (5.7)	12 (3.8)	30 (9.5)

## Discussion

Participants who are not in the target group for the Danish National CRC screening had a higher proportion of false-positive screening compared to the average-risk population. The odds of having a false-positive screening result were statistically significantly higher among all non-average risk groups, except for participants with multiple/hereditary polyps.

A major strength of this study is the use of high-quality Danish registry data, the registries have shown to have a high patient and data completeness, which minimizes the risk of selection and information bias [22, 26–28]. The use of registry data ensured the inclusion of all participants with a positive index FIT in the CRC screening program in 2014–2017.

We only included participants who attended the colonoscopy within three months. This criterion was established to ensure that the performed colonoscopy was screen-derived. In Denmark, it is mandatory for participants with a positive FIT to be invited to a colonoscopy within two weeks [4, 29], wherefore the application of this criterion is expected to have resulted in the exclusion of only a small proportion of participants and thus could only have had a minor effect on the outcome of the study.

When estimating the odds of having a false-positive screening we excluded participants with unknown registration of the colonoscopy outcome. We also excluded participants, who are not in the target group for FIT screening than average-risk participants. Even if all the participants with CRC, UC, CD or both UC and CD who were not registered, had been registered with positive colonoscopy, the adjusted odds of false-positive screening among these participants would still have been significantly higher than among the average-risk participants (Tables S1 and S2 placed in Supplementary Material). The elevated lack of colonoscopy outcome registration of IBD participants, i.e. no malignant, pre-malignant or clean colon codes may be due to registration related to the disease, which is not included in this study. A colonoscopy of individuals with IBD should be carried out with the dye Indigo carmine throughout the colon, or alternatively, a colonoscopy with white light to achieve the best result [15]. This might not be considered during in the organized screening program, which may affect the outcome among IBD participants.

It was not possible to collect information on the disease activity and phenotype among IBD participants, as such information is not in the registers. The categorization of IBD participants was based on their first registration with IBD in DNPR. The validity of this categorization would be higher if we had used two independent registrations [30, 31]. We defined participants with one IBD code as having IBD in order not to have IBD patients in the average group [4]. When we used two independent registrations, the odds of a false-positive screening test among IBD participants relative to average-risk participants was slightly higher for participants with UC (OR adjusted: 3.95 [95%CI: 3.35;4.67)] and CD [ORadjusted:4.09 (95%CI: 2.87:5.83)]. The exposure groups with UC and CD include participants with PSC, who have a higher risk of CRC [10]. They were included in the respective group as the numbers were too small to be reported separately. Due to the small numbers, it did not affect the result (data not shown).

When we changed the definition of false-positive screening to include both participants with clean colon and low-risk adenomas, 58.7% of the participants were categorized with a false-positive screening, and participants with CRC and IBD have a significant higher proportion of false-positive screening cases compared to the average risk population.

We are aware that some individuals with a false-positive screening may actually have had a false negative result in their screening-derived colonoscopy. While we believe the number of these cases is likely to be small and wouldn't significantly affect our results. However, we considered it relevant to uncover the proportion of false-negative screen-derived colonoscopies among individuals who are not in the target group for CRC screening.

The overall proportion of participants who attended the subsequent colonoscopy as well as the proportion of false-positive screening among average-risk participants are in line with previous Danish studies [14, 32, 33]. Likewise, the higher proportion of men with a true-positive screening, compared to the proportion with a false-positive screening is in line with previous systematic review, which included studies with cut-off values varying from 10 to 50 µg haemoglobin/g faeces [34]. A higher proportion of average-risk participants attended the colonoscopy compared to the other groups. Some of the participants not recommended to participate in FIT screening may have talked with their doctor after their positive FIT and afterward decided not to participate further in the screening program. The initial participation in FIT screening not followed by a subsequent colonoscopy is an unnecessary waste of resources.

It is unknown why 444 individuals with a previous CRC diagnosis participated in FIT screening, and 361 subsequently participated in the colonoscopy, when they should already be in a surveillance program. Some might be hoping for a negative FIT and thereafter settling with this, instead of participating in the colonoscopy surveillance program. It is also possible that some individuals with previous CRC worry about recurrence and therefore want to be checked more frequently [35]. It was not possible to compare the false-positive screening rate within the CRC group with other studies, as this group is excluded in similar studies [16, 34].

The distribution of UC and CD among participants with IBD, as well as the inclusion of more women than men with CD is consistent with the literature [36]. An earlier study from the German population-based screening colonoscopy program also found that individuals with IBD had a higher risk of a false-positive test than the average-risk population [16]. This probably reflects that some individuals with active IBD choose to participate in the screening programme, despite being recommended not to participate [15]. Active IBD in individuals with UC can manifest by bleedings from the gut [37], which could explain the higher proportion of false-positive screening among participants with UC compared with CD.

### Implications

The incidence and prevalence of IBD in Denmark are increasing, and the population with an IBD diagnosis is shifting towards an older age [36]. In the future, there will therefore be more individuals with IBD in the target age for FIT screening, which is why it is relevant to ensure clear recommendations for this group. The research team finds it relevant to study whether participants with IBD and previous CRC have been recommended to participate by their doctor. A qualitative study is likely to uncover why these participants decided to participate or not to participate in FIT screening. This knowledge is relevant for updating the information to individuals with IBD and previous CRC in the invitations letter. This study finds a higher risk of false-positive screenings among participants with IBD, but we do not find a higher proportion of detected CRC within this group. This might be due to CRCs detected during surveillance colonoscopies or fewer IBD patients attending screening with symptoms. The results of this study suggest that the Danish society performs too many screening-related colonoscopies in this group. With the knowledge that participants with a false-positive screening may experience negative psychosocial consequences [33], new and clear information in the invitations-procedures would be appropriate to make it easier for individuals who are not in the target population, to understand how they should proceed when they receive an invitation to the national screening program. This would help avoid redundant procedures, as FIT screening is not intended to be used as part of the monitoring process.

## Conclusions

This register-based cohort study shows that participants who are not in the target group for the Danish National FIT screening programme have a higher proportion of false-positive screening cases compared to the average-risk population when they choose to participate in CRC screening. The results of this study indicate that too many screen-derived colonoscopies are performed, which is why the research team recommends updating the screening invitation procedure.

## Supplementary Information

Below is the link to the electronic supplementary material.

Supplementary file1 (DOCX 20 KB)

## Data Availability

The data that support the result of this study are available from the Danish Colorectal Cancer Screening Database (DCCSD), the Danish Colorectal Cancer Group (DCCG) Database and the Danish Health Data Authority. Restrictions apply to the availability of the data, which were used under license for this study, and so not publicly available. Data may be available upon request.
