# Supplementary material for: Screening participants with inflammatory bowel disease or high colorectal cancer risk in Denmark: a cohort study
Source: J Public Health Policy. 2024 Oct 16;45(4):727–39. doi: 10.1057/s41271-024-00523-z (PMC11609084; doi:10.1057/s41271-024-00523-z)
Supplement: Supplementary file 1 — Supplementary file1 (DOCX 20 KB) [file 41271_2024_523_MOESM1_ESM.docx]

*Original Article*

Screening participants with inflammatory bowel disease or high colorectal cancer risk in Denmark: a cohort study

Signe Bülow Therkildsen^1^, Pernille Thordal Larsen^1,2^, Sisse Helle Njor*^1,2,3^

1 University Research Clinic for Cancer Screening, Department of Public Health Programmes, Randers Regional Hospital, Skovlyvej 15, 8930 Randers NØ, Denmark

2 Department of Clinical Medicine, Aarhus University, Palle-Juul-Jensen Boulevard 82, 8200 Aarhus N, Denmark

3 Research unit for screening and epidemiology, Department for Biochemistry and Immunology,
University Hospital of Southern Denmark, Beriderbakken 4, 7100 Vejle, Denmark

# **Supplementary Material**

**Table S1: Proportion and odds of false-positive relative to average-risk participants**

| Group | **Attended colonoscopy**  N** | **False positive**  n(%) | **OR**  (95%CI) | **Adjusted*** OR(95%CI) |
| --- | --- | --- | --- | --- |
| Average risk | 69.892 | 25.435 (36.4) | 1 | 1 |
| Prior colorectal cancer | 361 | 155 (42.9) | 1.32(1.07;1.62) | 1.55(1.25;1.91) |
| Multiple/hereditary polyp | >40 | 12 (NA) | 0.54(0.28;1.03) | 0.62(0.32;1.20) |
| Ulcerative colitis | 1.100 | 712 (64.7) | 3.21(2.83;3.63) | 3.12(2.75;3.66) |
| Crohn's disease | 317 | 184 (58.0) | 2.42(1.93;3.02) | 2.19(1.74;2.75) |
| Ulcerative colitis and Crohn's disease | 150 | 93 (62.0) | 2.85(2.05;3.97) | 2.66(2.90;3.73) |

** Adjusted for sex and five-year age groups*

*** Participants with unknown registration of colonoscopy outcome have been registered with a true positive colonoscopy, regardless of the exposure group (average-risk, previous CRC, prior CRC, Multiple/hereditary polyp syndromes, UC, CD or both UC and CD)*

**Table S2: Proportion and odds of false-positive relative to average-risk participants**

| **Group** | **Attended colonoscopy**  N** | **False positive**  n(%) | **OR**  (95%CI) | **Adjusted*** OR(95%CI) |
| --- | --- | --- | --- | --- |
| Average risk | 69.892 | 27.186 (38.9) | 1 | 1 |
| Prior colorectal cancer | 361 | 155 (42.9) | 1.18(0.96;1.46) | 1.38(1.12;1.71) |
| Multiple/hereditary polyp | >40 | 12 (NA) | 0.48(0.25;0.92) | 0.56(0.29;1.08) |
| Ulcerative colitis | 1.100 | 712 (64.7) | 2.88(2.54;3.27) | 2.80(2.47;3.18) |
| Crohn's disease | 317 | 184 (58.0) | 2.17(1.74;2.72) | 1.95(1.56;2.45) |
| Ulcerative colitis and Crohn's disease | 150 | 93 (62.0) | 2.56(1.84;3.57) | 2.38(1.70;3.34) |

** Adjusted for sex and five-year age groups*

*** All the participants in the high-risk groups (previous CRC, prior CRC, Multiple/hereditary polyp syndromes, UC, CD or both UC and CD) without a registration of the colonoscopy outcome have been registered with a true positive colonoscopy whereas average-risk participants have been registered with a negative colonoscopy.*
